# Supplementary material for: Reverse dynamic nuclear polarisation for indirect detection of nuclear spins close to unpaired electrons
Source: Magn Reson (Gott). 2022 Aug 10;3(2):161–8. doi: 10.5194/mr-3-161-2022 (PMC10539835; doi:10.5194/mr-3-161-2022)
Supplement: The supplement related to this article is available online at: https://doi.org/10.5194/mr-3-161-2022-supplement. [file mr-3-161-supplement.pdf]

Supplement of Magn. Reson., 3, 161–168, 2022
https://doi.org/10.5194/mr-3-161-2022-supplement
© Author(s) 2022. CC BY 4.0 License.

Supplement of

Reverse dynamic nuclear polarisation for indirect detection of nuclear spins close to unpaired electrons

Nino Wili et al.

Correspondence to: Nino Wili (nino.wili@alumni.ethz.ch)

The copyright of individual parts of the supplement might differ from the article licence.

Contents

S 1 Structure and EPR spectrum of OX063 **2**

S 2 Off-resonance transients in depolarisation curves **3**

S 3 Nutation curves and spectra **4**

S 4 Resonator Profile **5**

S 5 Details about RA-NOVEL **6**

S 6 Comparison of $T_{1,e}$ **7**

S 7 Comparison of $T_{1,\rho}$ **8**

S 8 Comparison of T_m **9**

S 9 Saturation behaviour during depolarisation **10**

S 10 Saturation behaviour in reverse DNP experiments **11**

S 11 Shots and averages per figure **13**

S 1 Structure and EPR spectrum of OX063

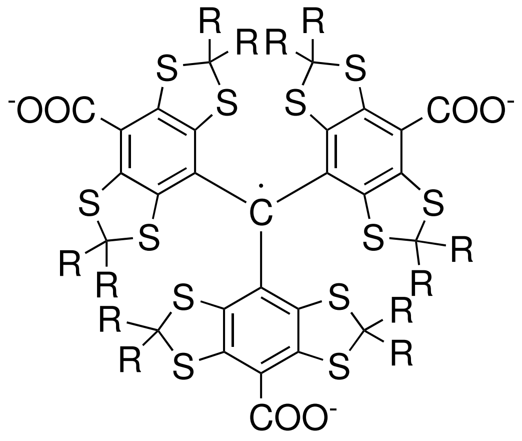

OX063 (R = CH_2CH_2OH)

Fig. S1: Chemical structure of the OX063 radical.

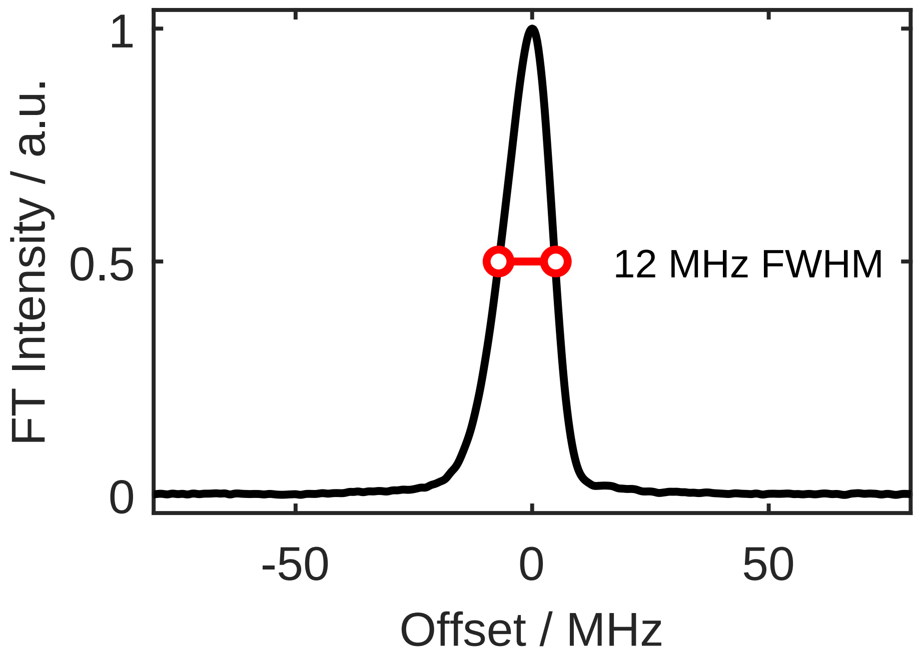

Fig. S2: Chirp echo Fourier transform EPR spectrum of OX063 obtained at a field of 1.2422 T.

S 2 Off-resonance transients in depolarisation curves

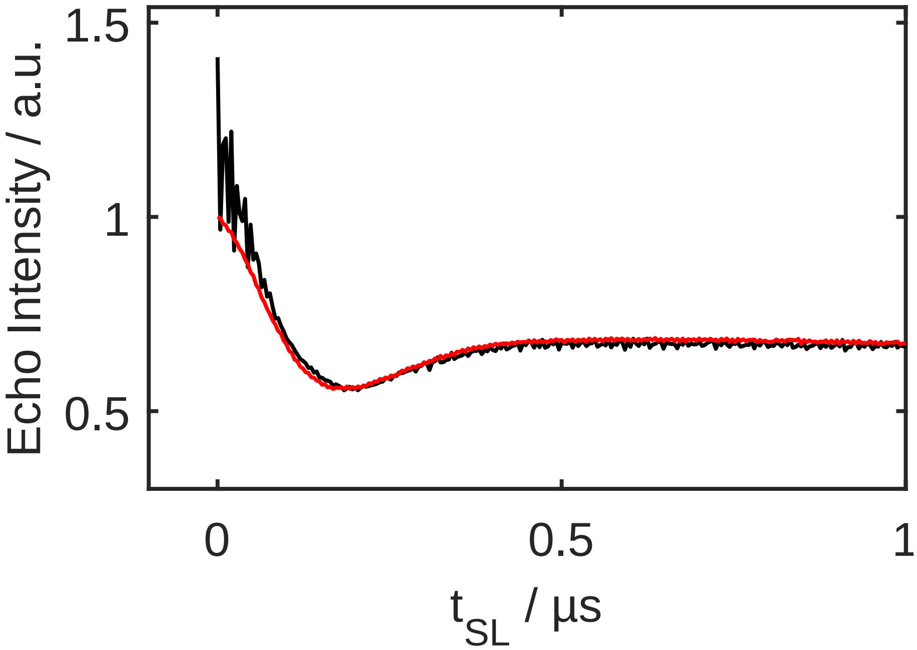

Fig. S3: Comparison of initial part of the depolarisation curve. Black: NOVEL condition fulfilled from the beginning of the spinlock, right after the initial $\pi/2$ pulse. Red: The first 500 ns of the spinlock used the maximum power, then it was dropped to the NOVEL condition ($t = 0$ corresponds to the time where the matching is fulfilled).

S 3 Nutation curves and spectra

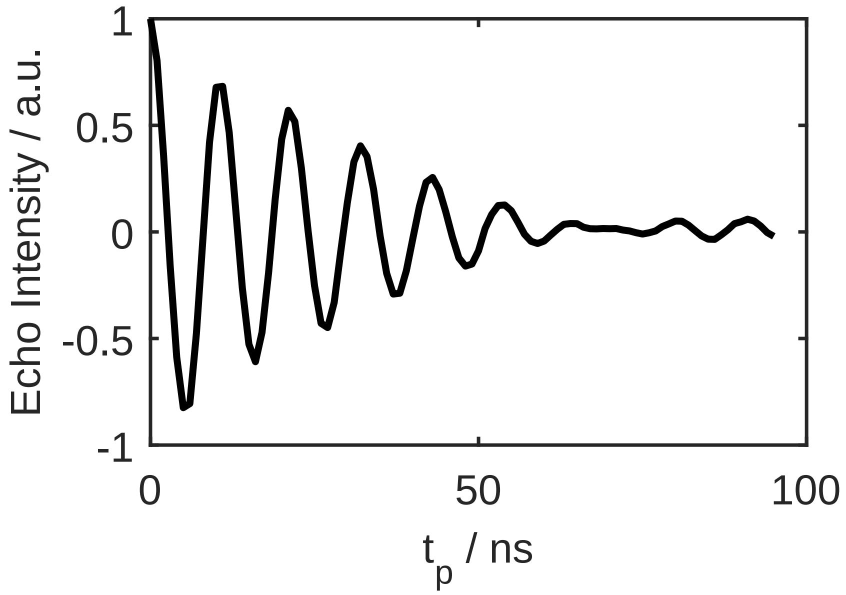

Fig. S4: Nutation curve obtained at the highest power with the sequence $t_{\mathrm{p}} - T - \pi/2 - \tau - \pi - \tau - echo$.

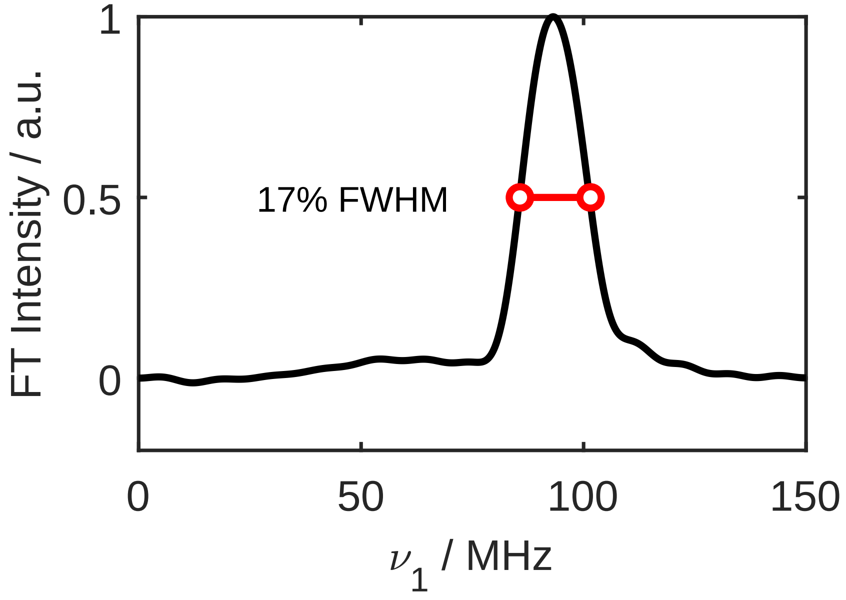

Fig. S5: Nutation spectrum, i.e. the Fourier Transform of Figure S4.

S 4 Resonator Profile

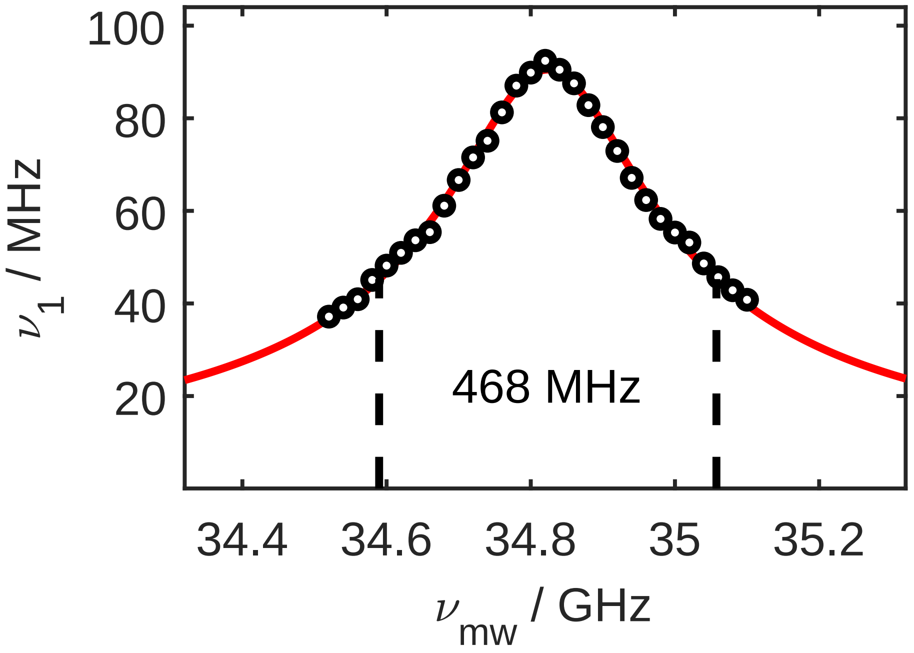

Fig. S6: Resonator profile at full mw power, obtained by measuring nutation spectra at different frequencies. The field was always set on resonance. The plotted value of ν_1 corresponds to the maximum of the nutation spectrum. The FWHM of the resonator profile is 468 MHz.

S 5 Details about RA-NOVEL

The following amplitude modulation function was used:

$$AM(x) = a_0 - \kappa \tan(2 \arctan(\Delta a/\kappa)(0.5 - x)) \quad (1)$$

with $x = t/t_{max}$, $a_0 = 0.27$, $\Delta a = 0.2$ and $\kappa = 0.025$. The scale corresponds to the digital output of the AWG. $a_0 = 0.265$ corresponded to the normal NOVEL condition. No compensation of the non-linearity of the TWT amplifier was used. When sweeping the pulse length, the waveform was simply stretched.

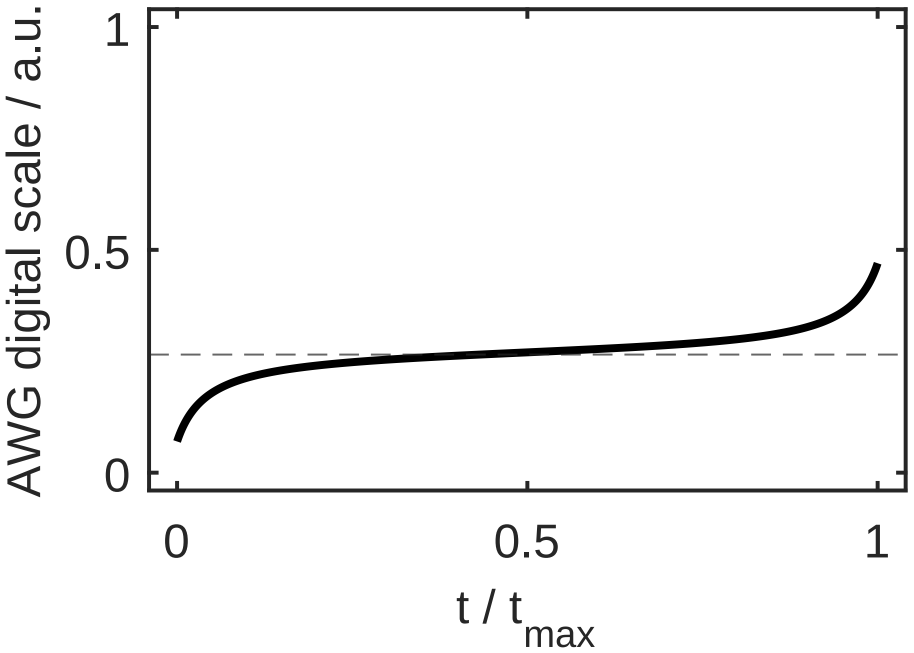

Fig. S7: Waveform used for RA-NOVEL experiments.

S 6 Comparison of $T_{1,e}$

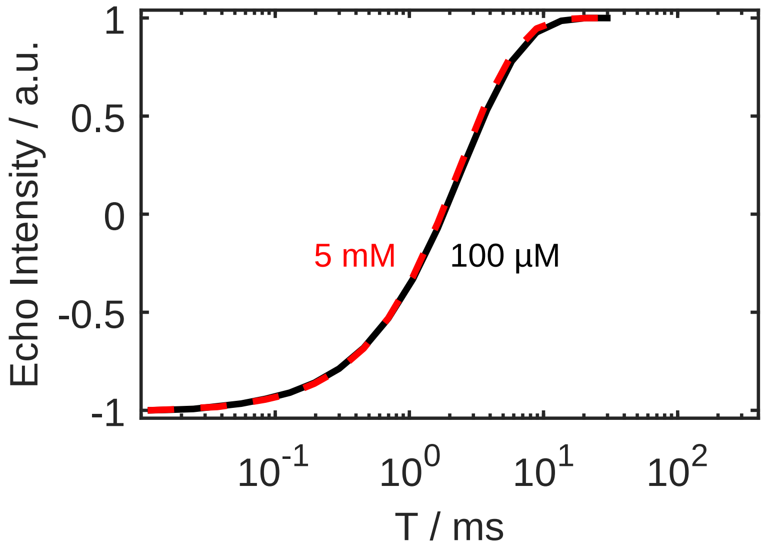

Fig. S8: Inversion recovery curves obtained at 80 K.

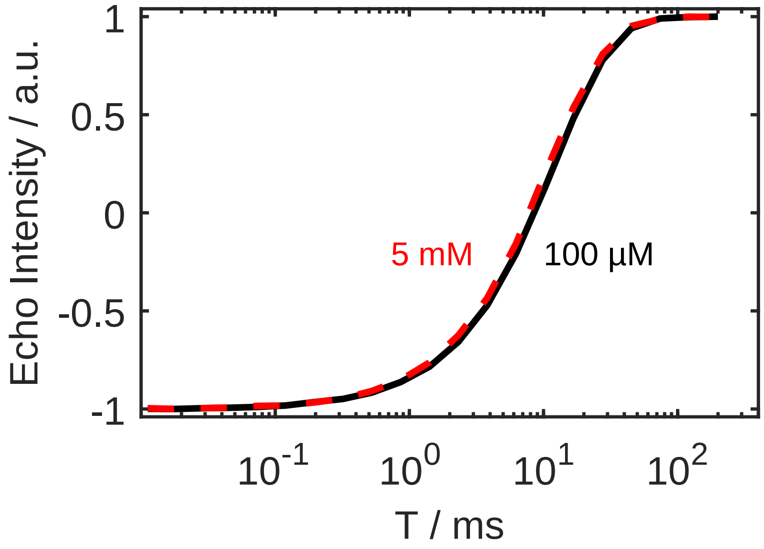

Fig. S9: Inversion recovery curves obtained at 50 K.

S 7 Comparison of $T_{1,\rho}$

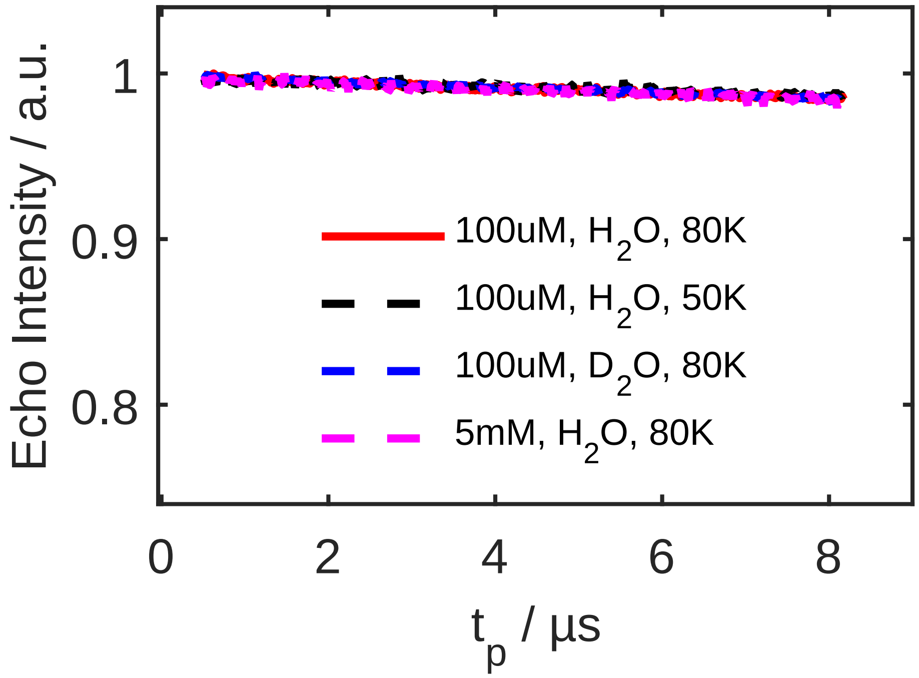

Fig. S10: Measurement of $T_{1\rho}$ for different samples and conditions. The spinlock power was set to the maximum. Since only a few percent of the signal are lost, and there is no difference between samples, the influence of $T_{1,\rho}$ can be neglected for the experiments presented in this work.

S 8 Comparison of T_m

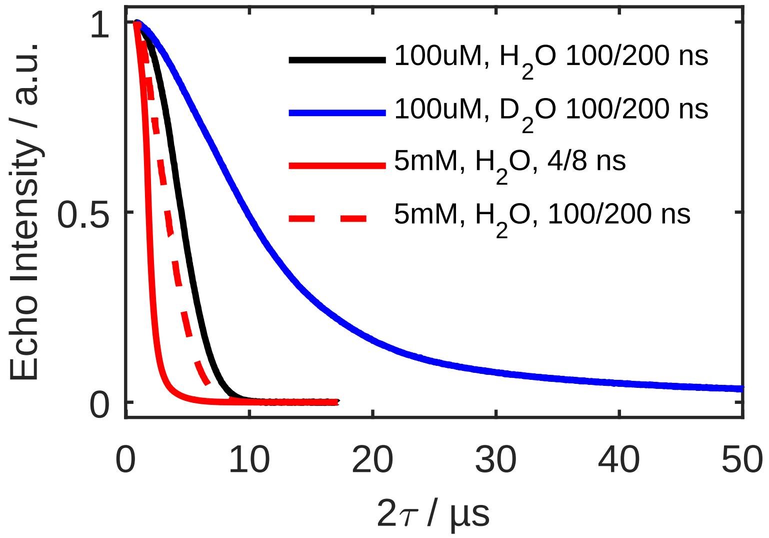

Fig. S11: Hahn echo decays measured at 80 K. the legend indicates the sample and the pulse lengths. As expected, deuteration of the solvent significantly increases the phase memory time. Additionally, the influence of instantaneous diffusion is pronounced at 5 mM. This can be seen because a shortening of the pulses, i.e. an increase in excitation bandwidth, leads to a faster Hahn echo decay.

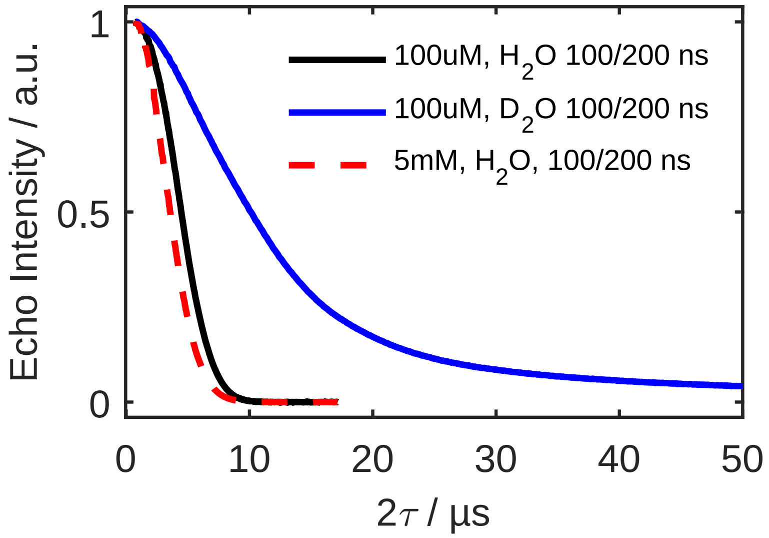

Fig. S12: Hahn echo decays measured at 50 K. There is no significant difference compared to 80 K.

S 9 Saturation behaviour during depolarisation

As shown in the main text, the nuclear relaxation is much slower than $T_{1,e}$. This raises the concern that saturation effects could appear when repeating experiments with the "normal" repetition times used for EPR experiments. To investigate this we performed a simple experiment: The pulse sequence in Figure 1(a) of the main text was repeated several times with a repetition time of 10 ms, with the spinlock fulfilling the NOVEL conditions for a duration of 500 ns. This should lead to an accumulation of nuclear polarisation, *if* no phase-cycling is used. Single echoes were detected, and the intensity normalised to the first echo. The results of this experiment are shown in Figure S13

In the case of deuterated solvent, there is only a small number of protons in the sample per electron. After a few DNP contacts, the protons are significantly polarized. Since the effective Hamiltonian during NOVEL leads to an oscillation of the *difference* of polarisations, less and less polarisation is transferred for each repetition, until some steady-state is reached. This leads to an apparent increase in Figure S13. In other words, less polarisation is lost from the electron to the nuclei during the spinlock. The electron polarisation does not rise over its equilibrium value.

In the case of the protonated solvent, no such "saturation effect" is observed, most likely because the amount of polarisation transferred per proton is so small and spin diffusion is fast enough that the difference between proton and electron spin polarisation is effectively constant over the timescale of the experiment. We did not check what happens if the experiment is repeated for several minutes.

Note that in the main text, all experiments are performed with phase-cycling. This leads to opposite nuclear polarisation for every other repetition, and thus nearly no net nuclear polarisation.

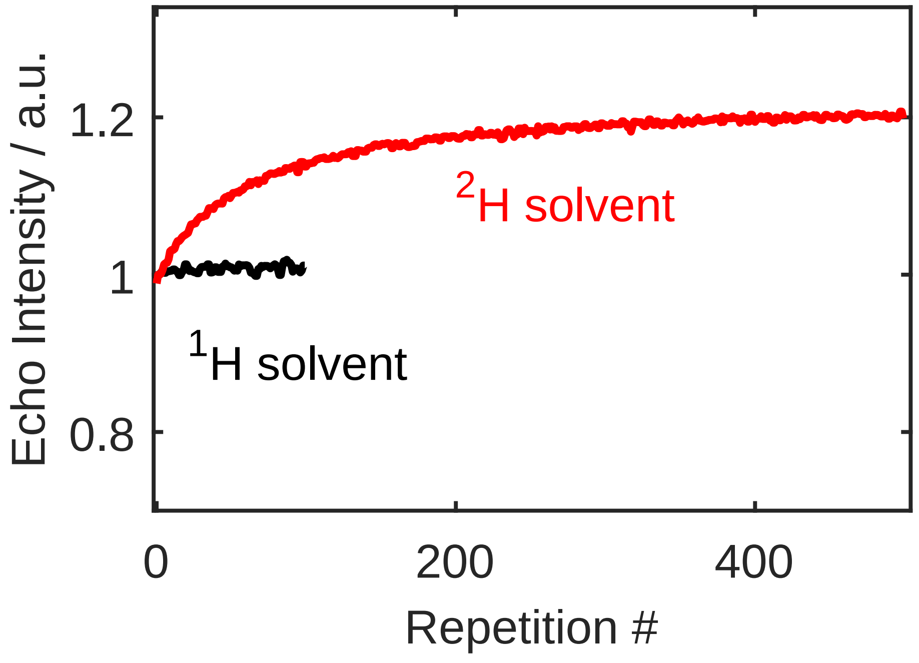

Fig. S13: Echo intensity after 500 ns of NOVEL matched spinlocking. Single echoes were detected without phase-cycling. 100 μM OX063.

S 10 Saturation behaviour in reverse DNP experiments

A significant nuclear polarisation at the beginning of the measurements might lead to unexpected effects during reverse DNP measurements. We cannot directly saturate the nuclei before, since we are currently using no rf-channel. Even if one would be available, directly coupled nuclei might be unaffected due to significant hyperfine coupling. However, saturating the electrons, then performing reverse DNP several times (see Figure S14) at least partially saturated the nuclei close to the electrons.

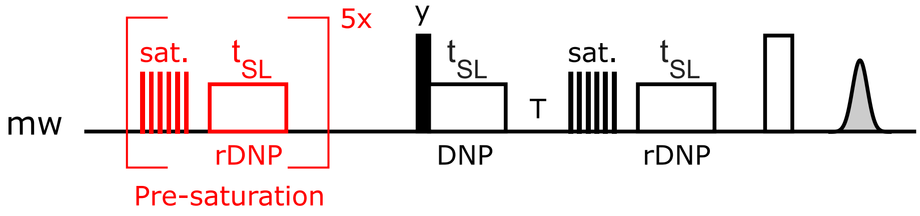

Fig. S14: Electron spin echo intensity after DNP and reverse DNP

The echo intensity after DNP and reverse DNP for the sample with deuterated solvent is shown in Figure S15. If no pre-saturation or phase-cycling is used, the intensity increases with each repetition, indicating an accumulation of nuclear polarisation. Presaturation alone reduces this accumulation, but is not sufficient to completely get rid of it. As expected, phase-cycling also does not lead to an accumulation of nuclear polarisation, because an opposite phase of the $\pi/2$ pulse leads to an opposite sign in nuclear polarisation. However, the first echo was always slightly more intense than the rest. If presaturation and phase-cycling are used together, the echo intensity is constant.

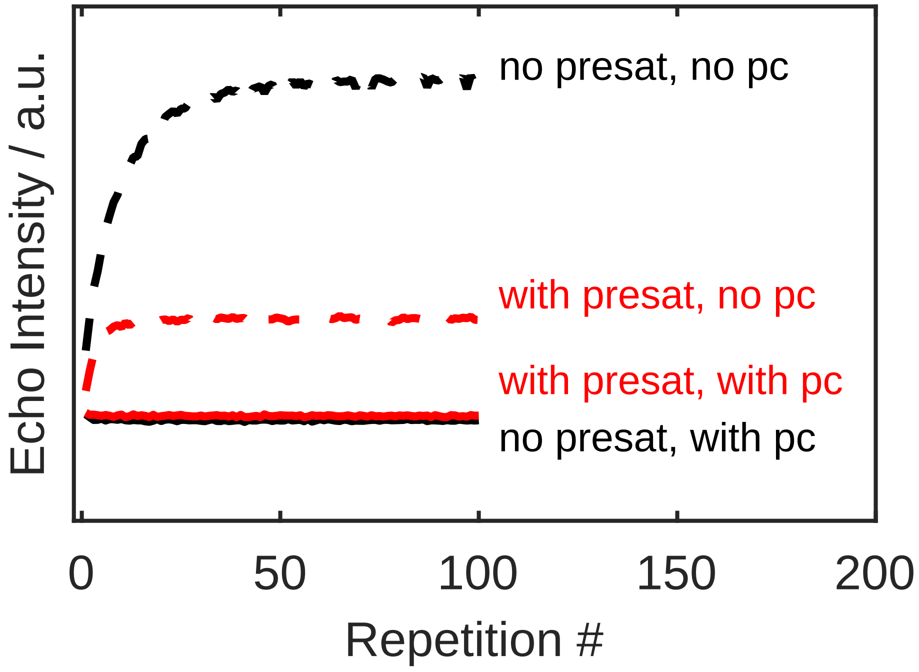

Fig. S15: Electron spin echo intensity after DNP and reverse DNP, using the 100 μM sample in deuterated solvent. The experiment was simply repeated several times, and single shots were acquired. A combination of presaturation and phase-cycling leads to results without any saturation behaviour from one repetition to the next.

S 11 Shots and averages per figure

Figure	Shots	Averages	Total time
1(b)	10	10	<1 min per line
1(c)	10	1	1–2 min
2(b) black	10	10	1–2 min
2(b) red	10	100	36 min
3(a)	10	1	1–2 min per line
3(b)	10	1	<1 min per line
4(a) red	20	1	1–2 min
4(a) black	5	1	1–2 min
4(b)	20	10	40 mins per line
5(a)	20	10	40 mins per line
5(c) w/o dec.	20	10	40 mins per line
5(c) with dec.	20	10	25 mins per line
